# Metabolically Healthy Obesity and Carotid Plaque among Steelworkers in North China: The Role of Inflammation

**DOI:** 10.3390/nu14235123

**Published:** 2022-12-02

**Authors:** Miao Yu, Shengkui Zhang, Lihua Wang, Jianhui Wu, Xiaoming Li, Juxiang Yuan

**Affiliations:** 1 Department of Epidemiology and Health Statistics, School of Public Health, North China University of Science and Technology, Tangshan 063210, China; 2 Department of Epidemiology and Statistics, Institute of Basic Medical Sciences, Chinese Academy of Medical, Beijing 100050, China

**Keywords:** metabolically healthy obesity, atherosclerosis, carotid plaque

## Abstract

This study aimed to investigate the association between metabolically healthy obesity (MHO) and carotid plaque. In this cross-sectional survey, 3467 steelworkers in North China were surveyed. There are two criteria for defining a carotid plaque: (1) the lesion structure exceeds 50% of the peripheral intima-media thickness value or invades the arterial lumen by at least 0.5 mm; (2) a thickness > 1.5 mm from the intima–lumen interface to the media–adventitia interface. Metabolic health was defined as the nonexistence of one of the metabolic syndrome (MetS) diagnostic criteria for metabolic abnormalities. Obesity was defined as having a BMI ≥ 25 kg/m^2^. To calculate the odds ratio (OR) for the prevalence carotid plaque, a logistic regression was used for the analysis. The prevalence of carotid plaque in the subjects was 14.3% for metabolically healthy non-obesity (MHNO), 32.4% for MHO, 18.9% for metabolically unhealthy non-obesity (MUNO), and 46.8% for metabolically unhealthy obesity (MUO). The odds ratios for suffering from carotid plaque were 1.27 (95% CI: 0.69 to 2.32) for MHO, 1.83 (95% CI: 1.29 to 2.58) for MUNO, and 1.81 (1.28 to 2.56) for MUO in comparison with MHNO after adjusting for confounders. There was no association between the MHO phenotype and carotid plaque prevalence among steelworkers in North China.

## 1. Introduction

In recent years, cardiovascular disease (CVD) has become one of the most deadly diseases in many countries around the world, with CVD accounting for 37% of the 17 million deaths of those under the age of seventy caused by noncommunicable diseases in 2019, which creates a huge burden worldwide [1]. In 2016, more than 2 million people died of atherosclerotic CVD (ASCVD) in China, accounting for 25% and 61% of all deaths and CVD deaths worldwide, respectively [2]. A research study of Chinese workplaces encapsulated that malignant tumors, CVD, respiratory diseases, and infectious diseases were the top four diseases threatening the workers’ lives, accounting for 73.2% of all worker deaths [3]. In addition, steelworkers had a higher prevalence of carotid plaque (31.7%) than the general population (21.1%) [4,5]. Such a high prevalence rate poses a serious health risk to steelworkers. Presently, a large proportion of adults with chronic conditions (e.g., CVD) are likely to have jobs, and thus, the associated risk factors and conditions are becoming more prevalent in the workforce [6]. In fact, changes in the body begin decades before the appearance of CVD. However, when CVD is used as the endpoint, the early changes in the worker’s body are not observed, which in turn affects the early prevention of CVD. Therefore, it is especially important to identify the causative factors in the etiological chain in the pre-disease stage. Carotid plaque, as morphological evidence of atherosclerosis, can predict CVD before the appearance of vascular symptoms and is a powerful predictor of CVD [7].

Obesity has been on the rise since 1980, and is now a global epidemic [8]. It is estimated that approximately 46% of adults in China are overweight or obese [9]. Worse still, studies have shown that obesity and hypertension are the leading causes of cardiovascular death among workers [10,11]. Obesity usually coexists with metabolic syndrome, which is often described as metabolically unhealthy obesity (MUO) [12]. It is well-known that early atherosclerosis can be accelerated by obesity [13]. Nevertheless, the risk of cardiometabolic disease is not increased in all obese people. Therefore, the concept of metabolically healthy obesity (MHO), an obese population without metabolic abnormalities, has been proposed by scholars and has recently received increasing attention [14]. This classification of the obesity phenotype has been widely used in many national populations [15,16,17]. Despite this, the connection between disease and obesity phenotypes remains controversial and is not well-studied.

A Korean community-based general population cohort study suggested that obesity increases the incidence of carotid plaque in metabolically healthy participants [18]. In contrast, there are also some different views. Two studies showed that the risk of carotid atherosclerosis was not increased by MHO compared to metabolically healthy non-obese individuals (MHNO) [19,20]. In summary, it is inconclusive whether MHO causes an increased risk of carotid plaque. Moreover, studies have shown that an inflammatory state is present in obesity and metabolic abnormalities such as elevated c-reactive protein [21,22]. Inflammation has also been linked to carotid plaque in several studies [23,24]. Thus, with a better understanding of obesity, metabolism and inflammation, obesity-related complications including cardiovascular disease can be better predicted.

To our knowledge, no previous studies have examined the relationship between different obesity phenotypes and carotid plaque by using imaging techniques with steelworkers. Therefore, the aim of this study was to investigate the association between MHO and the prevalence of carotid plaque among steelworkers in northern China, and the effect of inflammation on this association.

## 2. Materials and Methods

### 2.1. Study Design and Population

**Figure 1:**
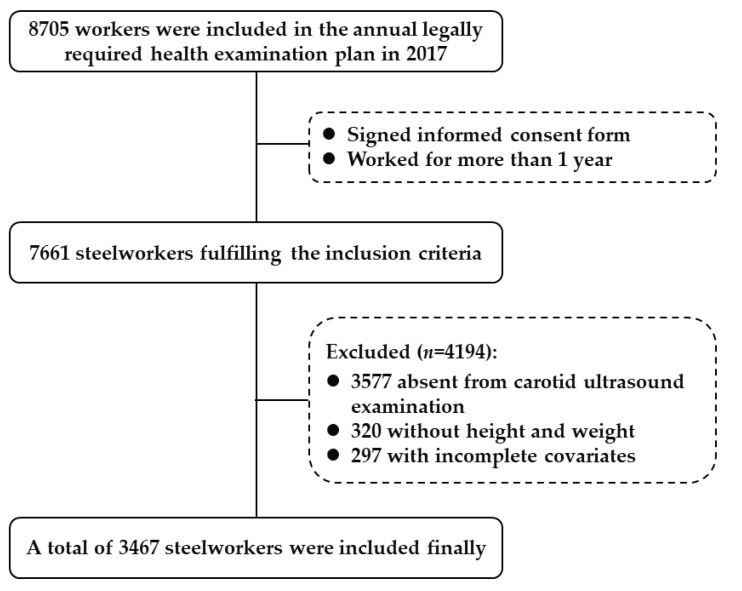
Flowchart of the participant selection.

This study was a cross-sectional study. The subjects of the study were steelworkers in 11 steel production departments of the Hebei Iron and Steel Group Tang Steel Company. The inclusion criteria were as follows: (1) participants who signed an informed consent form; and (2) participants who worked for more than 1 year. A total of 7661 steelworkers were recruited from February to June 2017. Our exclusion criteria were as follows: (1) participants who did not undergo carotid ultrasound examination; and (2) missing vital data such as height, weight, and covariates. Ultimately, 3467 steelworkers were included in our study (Figure 1). Ethical approval was given by the Ethics Committee of North China University of Science and Technology (No: 16040). All participants signed an informed consent form prior to participation in the study.

### 2.2. Definitions

The definitions of obesity, metabolic abnormalities, and obesity phenotypes have been detailed in our previous studies [25]. In brief, obesity was defined as a body mass index (BMI) ≥ 25 kg/m^2^ in accordance with the obesity diagnostic criteria established by the World Health Organization for Asian populations [26,27]. Several previous studies have used this definition and have confirmed its validity [28,29]. Metabolic health is defined as the non-existence of one of the metabolic syndrome (MetS) diagnostic criteria for metabolic abnormalities. Furthermore, according to the median value of the high-sensitivity c-reactive protein (hs-CRP), participants were divided into two groups [28].

Carotid plaque in the right and left carotid system was assessed by using a high-resolution b-mode ultrasound system (PHILIPS, HD7, Suzhou, China), which was performed by two sonographers who were trained. The sonographers were unaware of the study objectives and study design pairs. In the evaluation of carotid plaque, participants remained with their neck fully extended in the direction of the probe rotation whilst in a supine position. There were two criteria for defining a carotid plaque: (1) the lesion structure exceeded 50% of the peripheral intima-media thickness (IMT) value or invaded the arterial lumen by at least 0.5 mm; and (2) a thickness > 1.5 mm from the media–adventitia interface to the intima–lumen interface [30,31].

### 2.3. Evaluation of Covariates

Detailed covariate assessments and definitions are provided in the Supplementary Material S1.

### 2.4. Statistical Analysis

The study subjects were characterized according to their carotid plaque status and obesity phenotype. Assuming a normal distribution, the means and standard deviations of the measures were calculated. The *t*-test or analysis of variance (ANOVA) was used to compare the differences between groups for the measurement data. In case the continuous variable was non-normally distributed, the Kruskal–Wallis test and median (upper quartile, lower quartile) were used for the description and comparison. Group comparisons were made using the χ^2^ test, and data were expressed as numbers and percentages.

The relationships between carotid plaque and different obesity phenotypes, obesity and metabolic indicators, and inflammation were analyzed via multifactorial logistic regression. The interaction between hs-CRP and the metabolic status on the odds of carotid plaque was presented as a relative excess risk due to interaction and the proportion attributable to the interaction. The association between obesity and metabolic indicators and the odds of carotid plaque was assessed by a restricted cubic spline curve (RCS) with three nodes. SAS V.9.4 (SAS Institute, Cary, NC, USA) was used to perform the data analysis. The significance level was set at *p* < 0.05 using a two-tailed test.

## 3. Results

### 3.1. General Characteristics of the Participants

In Table 1, the general characteristics of the included 3467 patients are demonstrated. The mean age of participants in this study was 46.0 years, and the prevalence of carotid plaque was 30.1%. As shown in Table 1, steelworkers with carotid plaque were more likely to be current smokers and current alcohol drinkers. The obesity rate for all participants was 49.9%. The prevalence of carotid plaque in the subjects was 14.3% for MHNO, 32.4% for MHO, 18.9% for MUNO, and 46.8% for MUO. Additionally, the essential features according to the obesity phenotype are demonstrated in Supplementary Table S1. The carotid plaque prevalence was 32.0% in men and 12.7% in women, as shown in Supplementary Table S2.

### 3.2. Carotid Plaque Risk in Obesity Phenotypes and Metabolic Status

**Figure 2:**
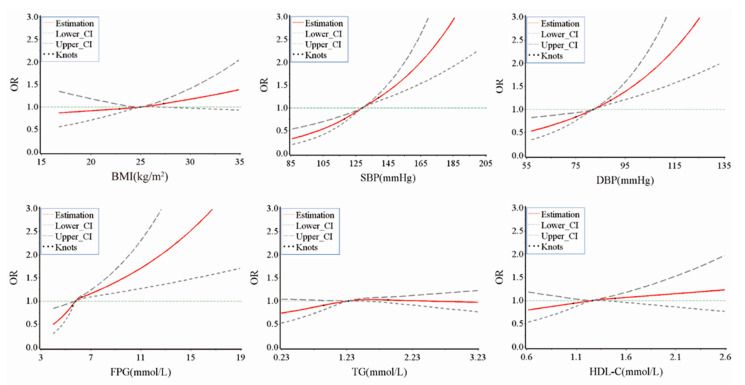
Association of the continuous metrics of BMI and metabolic indicators and the odds of carotid plaque from RCS models. Point estimates of OR or difference, upper and lower 95% Cis, and knots are indicated by red solid lines, grey long dashed lines, grey short dashed lines, and green dots respectively. Adjusted for age, sex, educational level, smoking status, drinking status, physical activity, DASH score, diabetes status, and hs-CRP. BMI, body mass index; SBP, systolic blood pressure; DBP, diastolic blood pressure; FBG, fasting blood glucose, TG, triglycerides; HDL-C, high-density lipoprotein cholesterol.

Carotid plaque was found in 14.3% (48/336) in the MHNO group, 32.4% (454/1402) in the MUNO group, 18.9% (20/86) in the MHO group, and 17.33% (522/1623) in the MUO group. MUNO (1.83 (95% CI: 1.29 to 2.58)) and MUO (1.81 (1.28 to 2.56)) were significantly related to carotid plaque risk compared to the MHNO controls. However, this relationship was not observed in the MHO (1.27 (0.69 to 2.32)) (Table 2). Participants with higher blood pressure levels (1.75 (1.48 to 2.07)) had a higher risk of carotid plaque compared to those with lower blood pressure levels. High levels of FBG (1.36 (1.13 to 1.63)), but not high TG and HDL-C were associated with carotid plaque. Compared with metabolic healthy individuals, the ORs (95% CI) for carotid plaque according to the metabolic abnormality component number were 1.43 (1.05 to 1.95) for one metabolic abnormality, 1.72 (1.27 to 2.34) for two metabolic abnormalities, 1.91 (1.37 to 2.65) for three metabolic abnormalities, and 2.02 (1.32 to 3.08) for four metabolic abnormalities (Table 3). MUNO and MUO were associated with carotid plaque irrespective of inflammatory status using the MHNO/hs-CRP ≤ 0.01 mg/dL group as a reference (Table 4). In addition, positive associations were observed between SBP (continuous), DBP (continuous), FBG (continuous), and the odds of carotid plaque (bivariate, yes/no) (Figure 2).

### 3.3. Sensitivity Analyses

In Supplementary Table S3, a potential interaction between hs-CRP and metabolic abnormalities is shown and multiplicative or additive interactions were not observed between hs-CRP and metabolic abnormalities with regard to carotid plaque prevalence (Supplementary Table S3). A stratified analysis explored the relationship between obesity phenotypes and carotid plaque under different stratification factors. The results show that participants in both thee smoking status groups and drinking status groups, the high physical activity group, and hs-CRP > 0.01 mg/dL group revealed a significant association between carotid plaque and the MUNO and MUO obesity phenotypes (Supplementary Table S4). As shown in Supplementary Table S5, we further adjusted for the main occupational disease hazards to which steelworkers are exposed including dust, high temperature, noise, and carbon monoxide based on the multivariate analysis, and the results remained robust. Moreover, in the sensitivity analyses (Supplementary Table S6), the association between the MHO phenotype and carotid plaque was largely unchanged when using the WHO-recommended international BMI cutoff points.

## 4. Discussion

In this cross-sectional study based on steelworkers, nearly half of the participants (49.9%) were obese, of which 5.0% was classified as “metabolically healthy” subjects, that is, had an MHO phenotype. The prevalence of MHO varies among studies, and has been reported to range from 4.2% to 13.6%, which is higher than the results of our study [32]. The present study found that MHO did not show a high risk for carotid plaque in any model compared with MHNO. However, both MUNO and MUO were associated with higher odds of carotid plaque in steelworkers compared to MHNO. These associations were supported by subgroup analyses. A further adjustment for hs-CRP reduced these correlations. Therefore, our results provide additional information showing that, even in the absence of obesity, only metabolic abnormality itself can lead to carotid plaque, and the association between carotid plaque and MUNO and MUO phenotypes may be explained in small part by an adverse inflammatory status.

Apart from this, the relationship between MHO and carotid plaque was inconsistent. A study in 980 Korean men showed that MHO was not associated with carotid plaque and carotid atherosclerosis [19]. Similarly, Da et al. showed no increased risk of atherosclerosis in participants with the MHO phenotype in a recent cohort study of 7824 community adults [20]. However, a community-based cohort study in Japan showed that subjects with the MHO phenotype had a substantially increased risk for carotid plaque compared to non-obesity subjects [18]. The reasons behind these conflicting results are still unclear, but different applications of BMI or waist circumference, different definitions of metabolic status, and carotid plaque may be responsible. In addition, the difference in obesity phenotype and carotid plaque correlation probably indicates heterogeneity in the association of the obesity phenotype with carotid atherosclerosis regression. This heterogeneity may explain some of the inconsistent results.

Based on a relatively large population of steelworkers, this study determined the presence of deleterious effects of metabolic abnormalities on carotid plaque after a full consideration of potential biases. Additionally, participants with the MHO phenotype were unaffected by the metabolic complications of obesity. Stated differently, for a given level of obesity, this risk appears to be substantially lower than expected [33,34]. On the other hand, participants with metabolically unhealthy phenotypes (MUNO and MUO) were found to have an increased risk for carotid plaque. Through further analysis, we found that elevated BP and FBG in the metabolic abnormalities group were associated with carotid plaque. The relationship between BP and carotid plaque may be mediated through a variety of biological mechanisms including endothelial dysfunction and metabolic pathways [35]. Hypertension accelerates the formation of atherosclerotic plaques by causing endothelial dysfunction, leading to thrombosis and vascular occlusion [36,37]. In addition, hypertension affects the formation of carotid plaque through a combined mechanism of metabolic pathways and genetic inheritance [38]. There may be multiple mechanisms involved in the association between FBG and carotid plaque. First, previous studies have shown that elevated blood glucose can trigger endothelial dysfunction and cause oxidative stress [39,40,41]. In addition, elevated blood glucose may enhance the activation of inflammatory cytokines [42]. Another finding of our study was that the more metabolic abnormalities present in the participants, the higher the risk of suffering from carotid plaque. This suggests that there may be interactions between the components of our metabolic abnormalities. The exact link between multiple metabolic abnormalities and carotid plaque needs to be explored in more studies in the future.

An accepted view in recent years is that carotid plaque is associated with inflammation, which may also be a negative consequence of exposure to obesity and metabolic abnormalities. Therefore, the role of hs-CRP has also been explored. A stratified analysis showed that both MUNO and MUO were associated with carotid plaque, despite inflammatory status. Concurrently, there was no interaction between hs-CRP and metabolic abnormalities. These results may be due to the fact that the current study has only one inflammatory marker, hs-CRP, which is an acute phase protein and does not fully reflect the true level of inflammation. In future studies, the role of other markers of inflammation in the association between different obesity phenotypes and carotid plaque needs to be investigated in depth.

The strengths of this study include the detailed lifestyle information used and the large number of participants. A few limitations should be mentioned. First, this study was a cross-sectional study and therefore the causal relationship between the obesity phenotypes and carotid plaque could not be determined. Second, the levels of insulin resistance or insulin secretion were not assessed, and thus, some participants without common major metabolic abnormalities but with isolated insulin resistance may have been overlooked. Therefore, we cannot deny that there may exist errors in the classification of the participants. Nevertheless, the metabolic indicators used in this study are common in clinical practice and the validity of this definition has previously been demonstrated [43]. Third, apart from hs-CRP, we lack data on other markers of inflammation. Finally, ultrasound is a technology-dependent examination; therefore, there may be differences between physicians when diagnosing structural abnormalities.

## 5. Conclusions

In conclusion, the MHO phenotype is not associated with carotid plaque among steelworkers, but the abnormal metabolic phenotype is associated with carotid plaque. In the prevention of carotid plaque, people with metabolic abnormalities, whether obese or not, should be given special attention.

## Figures and Tables

**Table 1:** Essential features based on carotid plaque.

Variables	Overall	Without Plaque	With Plaque	*p* Value
	*n* = 3467	*n* = 2423	*n* = 1044	
Age (years), *n* (%)				<0.001
22–39	726 (20.9)	654 (27.0)	72 (6.9)	
40–49	1425 (41.1)	1056 (43.6)	369 (35.3)	
50–60	1316 (38.0)	713 (29.4)	603 (57.8)	
Education level, *n* (%)				<0.001
Primary or Middle	1021 (29.5)	598 (24.7)	423 (40.5)	
High school or college	1827 (52.7)	1308 (54.0)	519 (49.17)	
University and above	619 (17.9)	517 (21.3)	102 (9.8)	
BMI (kg/m^2^)				0.120
<25	1738 (50.1)	1236 (51.0)	502 (48.1)	
≥25	1729 (49.9)	1187 (49.0)	542 (51.9)	
Diabetes status				<0.001
No, *n* (%)	3000 (86.5)	2165 (89.4)	835 (80.0)	
Yes, *n* (%)	467 (13.5)	258 (10.6)	209 (20.0)	
Lifestyle factors				
Smoking status, *n* (%)				<0.001
Never/Ever	1665 (48.0)	1240 (51.2)	425 (40.7)	
Current	1802 (52.0)	1183 (48.8)	619 (59.3)	
Drinking status, *n* (%)				<0.001
Never/Ever	2139 (61.7)	1599 (66.0)	540 (51.7)	
Current	1328 (38.3)	824 (34.0)	504 (48.3)	
Physical activity, *n* (%)				0.901
Low/Moderate	282 (8.2)	198 (8.2)	84 (8.0)	
High	3185 (91.9)	2225 (91.8)	960 (92.0)	
DASH score, mean (SD)	21.6 (2.4)	21.6 (2.3)	21.6 (2.5)	0.793
Blood pressure (mmHg)				
SBP, mean (SD)	129.5 (16.5)	127.1 (15.6)	135.3 (17.2)	<0.001
DBP, mean (SD)	82.8 (10.6)	81.5 (10.3)	85.8 (10.8)	<0.001
Age (years), mean (SD)	46.0 (7.9)	44.3 (8.0)	49.9 (5.9)	<0.001
BMI (kg/m^2^), mean (SD)	25.2 (3.3)	25.1 (3.3)	25.4 (3.2)	0.014
Lipid profiles (mmol/L)				
TC, mean (SD)	5.2 (1.0)	5.0 (0.9)	5.4 (1.0)	<0.001
TG, median (IQR)	1.3 (0.9–1.9)	1.3 (0.9–1.10)	1.4 (0.9–2.0)	0.003
HDL-C, mean (SD)	1.3 (0.3)	1.3 (0.3)	1.4 (0.3)	0.007
LDL-C, mean (SD)	3.3 (0.9)	3.1 (0.8)	3.5 (0.9)	<0.001
FBG (mmol/L), mean (SD)	6.1 (1.4)	6.0 (1.2)	6.4 (1.7)	<0.001
hs-CRP (mg/dL), median (IQR)	0.01 (0.00–0.07)	0.01 (0.00–0.06)	0.02 (0.00–0.10)	<0.001

BMI, body mass index; DASH, dietary approaches to stop hypertension; SBP, systolic blood pressure; DBP, diastolic blood pressure; FBG, fasting blood glucose, TC, total cholesterol; TG, triglycerides; HDL-C, high-density lipoprotein cholesterol; LDL-C, low-density lipoprotein cholesterol; hs-CRP, high-sensitivity C-reactive protein; IQR, inter quartile range.

**Table 2:** Multifactorial logistic regression analyses for carotid plaque according to the obesity phenotypes.

	MHNO	MUNO	MHO	MUO
Cases/number (%)	48/336 (14.3)	454/1402 (32.4)	20/86 (18.9)	522/1623 (46.8)
Model 1	1.00	2.87 (2.08 to 3.98)	1.40 (0.79 to 2.48)	2.85 (2.06 to 3.93)
Model 2	1.00	2.01 (1.43 to 2.82)	1.28 (0.70 to 2.33)	2.07 (1.47 to 2.90)
Model 3	1.00	1.94 (1.38 to 2.74)	1.30 (0.71 to 2.39)	2.02 (1.44 to 2.84)
Model 4	1.00	1.83 (1.29 to 2.58)	1.27 (0.69 to 2.32)	1.81 (1.28 to 2.56)

Model 1 unadjusted. Model 2 was further adjusted for age, sex. Model 3 was further adjusted for educational level, smoking status, drinking status, physical activity, DASH score, and diabetes status. Model 4 was further adjusted for hs-CRP. MHNO, metabolically healthy non-obesity; MUNO, metabolically unhealthy non-obesity; MHO, metabolically healthy obesity; MUO, metabolically unhealthy obesity.

**Table 3:** Multifactorial logistic regression analyses for carotid plaque according to BMI, metabolic abnormality components, and metabolically healthy status.

	Model 1	Model 2	Model 3	Model 4
BMI (kg/m^2^)				
<25	1.00	1.00	1.00	1.00
≥25	1.12 (0.97 to 1.30)	1.11 (0.95 to 1.30)	1.08 (0.93 to 1.30)	1.05 (0.90 to 1.23)
Metabolic abnormality components				
BP	2.29 (1.95 to 2.67)	1.87 (1.59 to 2.21)	1.76 (1.49 to 2.09)	1.75 (1.48 to 2.07)
FBG	1.87 (1.59 to 2.21)	1.48 (1.24 to 1.77)	1.37 (1.14 to 1.64)	1.36 (1.13 to 1.63)
TG	1.18 (1.02 to 1.38)	1.15 (0.98 to 1.36)	1.08 (0.91 to 1.27)	1.06 (0.89 to 1.25)
HDL-C	0.74 (0.61 to 0.91)	0.85 (0.68 to 1.05)	0.85 (0.68 to 1.05)	0.83 (0.67 to 1.02)
Metabolic abnormality number				
0	1.00	1.00	1.00	1.00
1	1.84 (1.37 to 2.47)	1.49 (1.10 to 2.03)	1.44 (1.06 to 1.97)	1.43 (1.05 to 1.95)
2	2.67 (2.01 to 3.56)	1.92 (1.42 to 2.59)	1.75 (1.29 to 2.37)	1.72 (1.27 to 2.34)
3	3.06 (2.26 to 4.15)	2.21 (1.61 to 3.05)	1.96 (1.41 to 2.71)	1.91 (1.37 to 2.65)
4	3.05 (2.26 to 4.53)	2.38 (1.57 to 3.61)	2.08 (1.37 to 3.17)	2.02 (1.32 to 3.08)

Model 1 unadjusted. Model 2 was further adjusted for age, sex. Model 3 was further adjusted for educational level, smoking status, drinking status, physical activity, DASH score, and diabetes status. Model 4 was further adjusted for hs-CRP. BMI, body mass index; BP, blood pressure; FBG, fasting blood glucose, TG, triglycerides; HDL-C, high-density lipoprotein cholesterol.

**Table 4:** Multifactorial logistic regression analyses for carotid plaque according to the obesity phenotypes and inflammation.

hs-CRP (mg/dL)	Obesity Phenotype	Carotid Plaque		OR (95% CI)
		No, (*n* (%))	Yes, (*n* (%))	
≤0.01	MHNO	212 (8.6)	31 (3.0)	1.00
≤0.01	MUNO	596 (24.6)	275 (26.3)	2.17 (1.42 to 3.31)
≤0.01	MHO	39 (1.6)	12 (1.2)	1.76 (0.80 to 3.88)
≤0.01	MUO	454 (18.7)	190 (18.2)	1.87 (1.21 to 2.88)
>0.01	MHNO	76 (3.1)	17 (1.6)	1.60 (0.81 to 3.15)
>0.01	MUNO	352 (14.5)	179 (17.2)	2.09 (1.35 to 3.25)
>0.01	MHO	47 (1.9)	8 (0.8)	1.24 (0.52 to 3.00)
>0.01	MUO	647 (26.7)	332 (31.7)	2.41 (1.58 to 3.67)

Adjusted for age, sex, educational level, smoking status, drinking status, physical activity, DASH score, diabetes status, and hs-CRP. MHNO, metabolically healthy non-obesity; MUNO, metabolically unhealthy non-obesity; MHO, metabolically healthy obesity; MUO, metabolically unhealthy obesity.

## Data Availability

The datasets generated and analyzed during this study are available from the corresponding author upon reasonable request.

## Supplementary Materials

The following supporting information can be downloaded at: https://www.mdpi.com/article/10.3390/nu14235123/s1, Supplementary Material S1: Assessment of covariates; Table S1: Basic characteristics according to obesity phenotypes; Table S2: Basic characteristics according to sex; Table S3: Multivariate adjusted odds ratios for the association between obesity phenotypes and carotid plaque, stratified by smoking status, drinking status, physical activity, and hs-CRP; Table S4: Multivariate adjusted odds ratios for the association between obesity phenotypes and carotid plaque, stratified by smoking status, drinking status, physical activity, and hs-CRP; Table S5: Independent effect of obesity phenotypes on carotid plaque after further adjustment for the main occupational hazards; Table S6: Sensitivity analyses of the association between the MHO (defined by WHO-recommended international BMI cutoff points) phenotypes and carotid plaque.
