# Supplementary material for: Metabolically Healthy Obesity and Carotid Plaque among Steelworkers in North China: The Role of Inflammation
Source: Nutrients. 2022 Dec 2;14(23):5123. doi: 10.3390/nu14235123 (PMC9740549; doi:10.3390/nu14235123)
Supplement: Supplementary file 1 [file nutrients-14-05123-s001.zip › nutrients-1986321-supplementary.pdf]

Supplementary Material S1: Assessment of covariates

Standard study protocols were used to train qualified physicians and nurses prior to this survey. Height and weight were measured three times each. The participants stood upright and barefoot in light clothes.

Blood pressure measurements were performed three times five-minute intervals using an electronic sphygmomanometer (OMRON, HBP-1100, China), and the participants were required to rest for more than ten minutes. Finally, the mean was obtained for analysis.

Participants were required to fast overnight before the health examination and blood collection. Participants' anterior elbow vein blood was collected and centrifuged at room temperature (3000 r/min, 15 minutes) immediately. All blood samples were tested in the central laboratory of Tangshan Hongci Hospital Laboratory using automatic biochemical analysers (Mindrary, BS-800, China) within four hours.

Smoking status was evaluated from self-reported information, mainly including the age at starting smoking and the number of cigarettes consumed per day, and was divided into never smokers (who had never smoked in their lifetime); ever smokers (who had quit cigarettes earlier than 12 months before) and current smokers (who had regularly consumed ≥1 cigarette/day for at least the past 12 months)[1].

Drinking status was evaluated from self-reported information, mainly including the amount and frequency of alcohol consumed per week and was divided into never drinkers (never or almost never drank alcohol in the past 12 months and had not drunk in most weeks in any past year); ever drinkers (did not drink alcohol in most weeks in the past 12 months but did so in some past year(s)) and current drinkers (drank alcohol usually at least once a week over the past 12 months)[2].

The calculation of metabolic equivalents was based on the International Physical Activity Questionnaire (IPAQ)[3]. The workers with metabolic equivalent task (MET) [min/week] values < 600, 600-3000 and > 3000 were classified as having a low, moderate, and high level of physical activity respectively.

Dietary patterns were assessed based on the DASH diet score[4]. Dietary patterns were assessed based on the DASH diet score, which was based on eight foods and nutrients that were either emphasized or deemphasized in the DASH-style diet[5]. Each component was scored from 1 to 5 points according to fifths of intake, with 5 being the best score for higher intake of vegetables, fruits, nuts and legumes, whole grains, and low fat dairy products and for lower intake of sugar sweetened drinks, red and processed meats, and sodium.

Assessment of main occupational hazards

Exposure to dust was defined as workers who may be exposed to productive dust (inorganic dust, organic dust or mixed dust) during production (GBZ/T 229.1–2010). The total dust in the air of workplace was collected at the breathing zone with a filter membrane, and it's concentration was calculated based on the increased weight of the filter membrane and the amount of gas collected. When the dust concentration in the air ≤50 mg/m^3, a filter membrane with a diameter of 37mm or 40mm was used, otherwise a filter membrane with a diameter of 75mm would be used (GBZ/T 192.1–2007)[6].

Exposure to high temperature (heat stress work) was defined as the average wet-bulb globe temperature (WBGT) index of the workplace is equal or greater than 25°C in the process of production (GBZ 2.2–2007)[7]. The WBGT index was measured by black-wet bulb globe thermometer. If there was no productive heat source in the workplace, three measuring points were selected to take the average value of WBGT index, while where there was a productive heat source, 3 to 5 measuring points were selected to take the average value of WBGT index. If the workplace was isolated into different thermal or ventilated environment, 2 measuring points were selected to take the average value of WBGT index (GBZ/T 189.7–2007)[8].

Exposure to industrial toxicant was defined as workers who may be exposed to a variety of harmful chemicals (the toxicant specifically refers to carbon monoxide in this population) during production (GBZ/T 229.2–2010)[9]. Carbon monoxide or carbon dioxide in the air of workplace was pumped into the Non-Dispersive Infrared-Ray (NDIR) analyzer and selectively absorbs their infrared rays. The concentration of carbon monoxide was determined according to the absorption value (GBZ/T 160.28–2004)[10].

Exposure to noise was defined as workers who exposed to a noisy environment where the 8h/d or 40h/week equivalent A-weighted sound pressure level is≥80dB, which may be harmful to health and hearing (GBZ/T 229.4–2012)[11]. The workplace production noise was measured by a sound level meter. If the distribution of sound field in the workplace was uniform (between-field difference of A-sound levels were less than 3dB(A)), three measuring points were selected to take the average value, otherwise it should be divided into several sound level areas. In each sound field, two measuring points were selected

to take the average value (GBZ/T 189.8–2007)[12].

1. Li X, Cui S, Wu J, Wang L, Yuan J: **Job category differences in the prevalence and associated factors of insomnia in steel workers in China**. *International journal of occupational medicine and environmental health* 2020, **33**(2):215-233.
2. Millwood IY, Walters RG, Mei XW, Guo Y, Yang L, Bian Z, Bennett DA, Chen Y, Dong C, Hu R *et al*: **Conventional and genetic evidence on alcohol and vascular disease aetiology: a prospective study of 500 000 men and women in China**. *Lancet (London, England)* 2019, **393**(10183):1831-1842.
3. Celis-Morales CA, Perez-Bravo F, Ibañez L, Salas C, Bailey MES, Gill JMR: **Objective vs. self-reported physical activity and sedentary time: effects of measurement method on relationships with risk biomarkers**. *PloS one* 2012, **7**(5):e36345.
4. Maskarinec G, Lim U, Jacobs S, Monroe KR, Ernst T, Buchthal SD, Shepherd JA, Wilkens LR, Marchand LL, Boushey CJ: **Diet Quality in Midadulthood Predicts Visceral Adiposity and Liver Fatness in Older Ages: The Multiethnic Cohort Study**. *Obesity (Silver Spring, Md)* 2017, **25**(8):1442-1450.
5. Fung TT, Chiuve SE, McCullough ML, Rexrode KM, Logroscino G, Hu FB: **Adherence to a DASH-style diet and risk of coronary heart disease and stroke in women**. *Archives of internal medicine* 2008, **168**(7):713-720.
6. **Determination of dust in the air of workplace. Part 1: Total dust concentration.** [http://niohp.chinacdc.cn/zyysjk/zywsbzml/201210/t20121012_70522.htm]
7. **Occupational exposure limits for hazardous agents in the workplace. Part 2: Physical agents** [http://niohp.chinacdc.cn/zyysjk/zywsbzml/201303/t20130329_79199.htm]
8. **Measurement of physical agents in workplace. Part 7: Heat Stress** [http://niohp.chinacdc.cn/zyysjk/zywsbzml/201210/t20121012_70527.htm]
9. **Classification of occupational hazards at workplaces. Part 2: Occupational exposure to chemicals** [http://niohp.chinacdc.cn/zyysjk/zywsbzml/201210/t20121012_70489.htm]
10. **Methods for determination of inorganic carbon compounds in the air of workplace** [http://niohp.chinacdc.cn/zyysjk/zywsbzml/201210/t20121015_70624.htm]
11. **Classification of occupational hazards at workplaces. Part 4: Occupational exposure to noise** [http://niohp.chinacdc.cn/zyysjk/zywsbzml/201307/t20130715_84934.htm]
12. **Measurement of physical agents in workplace. Part 8: Noise** [http://niohp.chinacdc.cn/zyysjk/zywsbzml/201210/t20121012_70526.htm]

Supplementary Table S1 Basic characteristics according to obesity phenotypes.

Variables	Total	MHNO	MUNO	MHO	MUO	*P* value
	n=3467	*n*=336	*n*=1402	*n*=106	*n*=1623	
Age (years), mean (SD)	46.0 (7.9)	42.8 (8.7)	47.1 (7.5)	43.1 (8.4)	46.0 (7.7)	<0.001
hs-CRP (mg/dl), median (IQR)	0.01 (0.00–0.07)	0.00 (0.00–0.02)	0.01 (0.00–0.04)	0.02 (0.00–0.05)	0.03 (0.01–0.12)	<0.001
DASH score, mean (SD)	21.6 (2.4)	21.9 (2.3)	21.7 (2.4)	21.4 (2.3)	21.5 (2.4)	0.016
Age (years), n (%)						<0.001
22–39	726 (20.9)	118 (35.2)	236 (16.8)	33 (31.1)	339 (20.9)	
40–49	1425 (41.1)	142 (42.3)	567 (40.4)	49 (46.2)	667 (41.1)	
50–60	1316 (38.0)	76 (22.6)	599 (42.7)	24 (22.6)	617 (38.0)	
Education level, n (%)						<0.001
Primary or Middle	1021 (29.5)	72 (21.4)	441 (31.5)	25 (23.6)	483 (29.8)	
High school or college	1827 (52.7)	164 (48.8)	755 (53.9)	53 (50.0)	855 (52.7)	
University and above	619 (17.9)	100 (29.8)	206 (14.7)	28 (26.4)	285 (17.6)	
Smoking status, n (%)						<0.001

Never/Ever	1665 (48.0)	186 (55.4)	684 (48.8)	58 (54.7)	737 (45.4)	
Current	1802 (52.0)	150 (44.6)	718 (51.2)	48 (45.3)	886 (54.6)	
Drinking status, n (%)						
Never/Ever	2139 (61.7)	259 (77.1)	836 (59.6)	80 (75.5)	964 (59.4)	<0.001
Current	1328 (38.3)	77 (22.9)	566 (40.4)	26 (24.5)	659 (40.6)	
Physical activity, n (%)						0.241
Low/Moderate	282 (8.1)	21 (6.2)	127 (9.1)	6 (5.7)	128 (7.9)	
High	3185 (91.9)	315 (93.8)	1275 (90.9)	100 (94.3)	1495 (92.1)	
Plaque, n (%)	1044 (30.1)	48 (14.3)	454 (32.4)	20 (18.9)	522 (32.2)	<0.001

Supplementary Table S2 Basic characteristics according to sex.

Variables	Total	Male	Female	*P* value
	***n*=3467**	***n*=3136**	***n*=331**	
Age (years), n (%)				<0.001
22–39	726 (20.9)	663 (21.1)	63 (19.0)	
40–49	1425 (41.1)	1201 (38.3)	224 (67.7)	
50–60	1316 (38.0)	1272 (40.6)	44 (13.3)	
Education level, n (%)				0.042
Primary or Middle	1021 (29.5)	943 (30.1)	78 (23.6)	
High school or college	1827 (52.7)	1641 (52.3)	186 (56.2)	
University and above	619 (17.8)	552 (17.6)	67 (20.2)	
BMI (kg/m^2), mean (SD)	25.2 (3.3)	25.4 (3.3)	23.7 (3.0)	<0.001
Lifestyle factors				
Smoking status, n (%)				<0.001
Never/Ever	1665 (48.0)	1365 (43.5)	300 (90.6)	
Current	1802 (52.0)	1771 (56.5)	31 (9.4)	
Drinking status, n (%)				<0.001
Never/Ever	2139 (61.7)	1830 (58.4)	309 (93.3)	
Current	1328 (38.3)	1306 (41.7)	22 (6.7)	
Physical activity, n (%)				0.987
Low/Moderate	282 (8.1)	255 (8.1)	27 (8.2)	
High	3185 (91.9)	2881 (91.9)	304 (91.8)	
DASH score, mean (SD)	21.6 (2.4)	21.5 (2.3)	22.7 (2.5)	<0.001
Blood pressure (mmHg)				
SBP, mean (SD)	129.5 (16.5)	130.3 (16.5)	121.9 (14.5)	<0.001
DBP, mean (SD)	82.8 (10.6)	83.3 (10.6)	77.7 (9.7)	<0.001
Age (years), mean (SD)	46.0 (7.9)	46.1 (8.1)	44.7 (5.4)	0.002
Lipid profiles (mmol/L)				
TC, mean (SD)	5.2 (1.0)	5.2 (1.0)	5.1 (1.0)	0.168
TG, median (IQR)	1.3 (0.9–1.9)	1.3 (0.9–2.0)	0.9 (0.7–1.3)	<0.001
HDL-C, mean (SD)	1.3 (0.3)	1.3 (0.3)	1.5 (0.4)	<0.001
LDL-C, mean (SD)	3.3 (0.9)	3.3 (0.9)	3.2 (0.9)	0.045
FPG (mmol/L), mean (SD)	6.1 (1.4)	6.2 (1.4)	5.8 (1.1)	<0.001
hs-CRP (mg/dl), median (IQR)	0.01 (0.00–0.07)	0.01 (0.00–0.07)	0.01 (0.00–0.04)	<0.001
Plaque, n (%)	1044 (30.1)	1002 (32.0)	42 (12.7)	<0.001

Supplementary Table S3 Multivariate adjusted odds ratios for the association between obesity phenotypes and carotid plaque, stratified by smoking status, drinking status, physical activity, and hs-CRP.

Interaction terms	Total
Multiplicative interaction, OR (95% CI)	2.01 (1.39 to 2.90)
Additive interaction [a]	
Relative excess risk due to interaction, RERI (95% CI)	-0.06 (-0.81 to 0.69)
Attributable proportion due to interaction, AP (95% CI)	0.03 (-0.40 to 0.34)
Synergy index, S (95% CI)	0.94 (-0.47 to 1.87)

OR, odds ratio; CI, confidence intervals; RERI, relative excess risk due to interaction; AP, attributable proportion due to interaction;

[a] If there is no biological interaction, RERI and AP are equal to 0 and S is equal to 1.

Adjusted for age, sex, educational level, smoking status, drinking status, physical activity, DASH score, BMI, and diabetes status.

Supplementary Table S4 Multivariate adjusted odds ratios for the association between obesity phenotypes and carotid plaque, stratified by smoking status, drinking status, physical activity, and hs-CRP.

	MHNO	MUNO	MHO	MUO	*p* for Interaction
Smoking status					0.054
Never/Ever	1.00	1.89 (1.12 to 3.20)	1.45 (0.61 to 3.45)	1.93 (1.13 to 3.30)	
Current	1.00	1.97 (1.25 to 3.10)	1.13 (0.48 to 2.65)	1.78 (1.13 to 2.82)	
Drinking status					0.001
Never/Ever	1.00	1.62 (1.07 to 2.46)	1.19 (0.57 to 2.47)	1.84 (1.21 to 2.81)	
Current	1.00	2.46 (1.33 to 4.56)	1.48 (0.50 to 4.39)	1.88 (1.01 to 3.50)	
Physical activity					0.71
Low/Moderate	1.00	1.00 (0.31 to 3.19)	0.70 (0.06 to 8.77)	1.08 (0.33 to 3.52)	
High	1.00	2.02 (1.41 to 2.90)	1.31 (0.70 to 2.45)	1.89 (1.31 to 2.46)	
hs-CRP (mg/dL)					0.078
≤0.01	1.00	2.25 (1.47 to 3.44)	1.73 (0.79 to 3.82)	1.93 (1.24 to 2.99)	
>0.01	1.00	1.37 (0.76 to 2.48)	0.77 (0.29 to 2.02)	1.45 (0.81 to 2.58)	

Adjusted for age, sex, educational level, smoking status, drinking status, physical activity, DASH score, diabetes status, hs-CRP.

Supplementary Table S5 Independent effect of obesity phenotypes on carotid plaque after further adjustment for the main occupational hazards.

Obesity phenotype	Plaque		OR (95% CI)
	No, (n (%))	Yes, (n (%))	
MHNO	288 (11.9)	48 (4.6)	1.00
MUNO	948 (39.1)	454 (43.5)	1.79 (1.26 to 2.55)
MHO	86 (3.6)	20 (1.9)	1.11 (0.60 to 2.06)
MUO	1101 (45.4)	522 (50.0)	1.77 (1.24 to 2.53)

Adjusted for age, sex, educational level, smoking status, drinking status, physical activity, DASH score, diabetes status, hs-CRP, dust exposure, heat stress exposure, noise exposure, and carbon monoxide exposure.

Supplementary Table S6 Sensitivity analyses of the association between MHO (defined by WHO-recommended international BMI cut-off points) phenotypes and carotid plaque.

Obesity phenotypes	Model 1	Model 2	Model 3	Model 4
Metabolic health				
MHN	1.00	1.00	1.00	1.00
MHOW	1.42 (0.78 to 2.58)	1.28 (0.68 to 2.38)	1.29 (0.69 to 2.42)	1.26 (0.67 to 2.37)
MHO	1.20 (0.26 to 5.65)	1.33 (0.27 to 6.60)	1.44 (0.29 to 7.15)	1.34 (0.27 to 6.62)

Metabolic unhealth				
MUN	2.87 (2.08 to 2.58)	2.00 (1.42 to 2.81)	1.94 (1.38 to 2.74)	1.83 (1.30 to 2.58)
MUOW	2.83 (2.04 to 3.98)	1.97 (1.40 to 2.78)	1.93 (1.37 to 2.73)	1.75 (1.24 to 2.49)
MUO	2.95 (1.98 to 4.40)	2.62 (1.72 to 4.00)	2.56 (1.67 to 3.91)	2.22 (1.44 to 3.42)

MHN, metabolically healthy normal weight; MHOW, metabolically healthy overweight; MHO, metabolically healthy obesity; MUN, metabolically unhealthy normal weight; MUOW, metabolically unhealthy overweight; MUO, metabolically unhealthy obesity.

Adjusted for age, sex, educational level, smoking status, drinking status, physical activity, DASH score, and hs-CRP, diabetes.

The classification of weight by BMI: normal weight (18.5-24.9 kg/m^2), overweight (BMI≥25.0–29.9 kg/m^2), obese (BMI≥30.0 kg/m^2).
